# Thanatochemical Study of Glycated Hemoglobin in Diabetic Status Assessment

**DOI:** 10.3390/medicina57040342

**Published:** 2021-04-02

**Authors:** Nona Girlescu, Bogdan Stoica, Andrei Daniel Timofte, Iuliana Hunea, Madalina Diac, Anton Knieling, Simona Irina Damian, Tatiana Iov, Diana Bulgaru Iliescu

**Affiliations:** 1 Morphofunctional Sciences 1 Department, Faculty of Medicine, “Grigore T. Popa” University of Medicine and Pharmacy of Iasi, 700115 Iasi, Romania; nona-girlescu@umfiasi.ro; 2 Institute of Forensic Medicine, 700455 Iasi, Romania; bogstoica@gmail.com (B.S.); zamisnicu.iuliana@gmail.com (I.H.); madalinadc89@gmail.com (M.D.); tony_knieling@yahoo.com (A.K.); si_damian@yahoo.com (S.I.D.); iovtatiana@yahoo.com (T.I.); bulgarudiana@yahoo.com (D.B.I.); 3 Morphofunctional Sciences 2 Department, Faculty of Medicine, “Grigore T. Popa” University of Medicine and Pharmacy of Iasi, 700115 Iasi, Romania; 4 Forensic Science Department, Faculty of Medicine, “Grigore T. Popa” University of Medicine and Pharmacy of Iasi, 700115 Iași, Romania

**Keywords:** thanatochemistry, glucose metabolism, glycated hemoglobin, diabetes, diagnostic

## Abstract

*Background and objectives.* In forensic medicine, the postmortem determination of glycated hemoglobin (HbA1c) helps identify undiagnosed cases of diabetes or cases with uncontrolled glycemic status. In order to contribute to the solidification of thanatochemistry, both globally and especially nationally, we aimed to determine this biomarker postmortem, for the first time in our institution, in order to identify undiagnosed pre-mortem diabetics, as well as those with inadequate glycemic control. *Materials and Methods.* Our research consisted of analyzing a total number of 180 HbA1c values, 90 determinations from the peripheral blood and 90 from the central blood. The determination of HbA1c was performed by means of a fully automatic analyzer (HemoCue HbA1c 501), certified by the National Glycohemoglobin Standardization Program (NGSP)/Diabetes Control and Complications Trial (DCCT) and calibrated according to the standards developed by the International Federation of Clinical Chemistry (IFCC). According to ADA criteria, HbA1c values can provide us with the following information about the diagnosis of diabetes: normal 4.8–5.6%; prediabetes 5.7–6.4%; diabetes ≥ 6.5%. *Results.* A considerable number of cases with an altered glycemic status (cases that had HbA1c values equal to or greater than 5.7%) were identified—51% demonstrable by peripheral blood determinations and 41% by central blood determinations. Notably, 23 people with diabetes (25%) were identified by analyzing the peripheral blood; 18 other people with diabetes (20%) were identified by analyzing the central blood. *Conclusions.* Our study managed to confirm the antemortem diagnosis of DM using a simple point-of-care analyzer and applying standardized and certified criteria on HbA1c levels measured postmortem. We also identified a considerable number of cases with DM in patients with no antemortem history of glucose imbalance—at least 20% more cases. Although the two different sites used for blood collection showed a strong statistical correlation, it seems that the peripheral site could have a higher sensibility in detecting postmortem altered glycemic status.

## 1. Introduction

During an autopsy, especially when the cause of death is suspected to be related to diabetes or alcohol consumption, complementary examinations, including those provided by thanatochemistry, are indispensable.

Glycated hemoglobin (HbA1c) is a well-known biomarker [1,2], along with others [3], used in the diagnosis and identification of people with a high risk of developing diabetes mellitus (DM) [1,2]. Since 2010, the International Expert Committee, the American Diabetes Association (ADA) and the World Health Organization (WHO) have introduced HbA1c as a diagnostic criterion for DM; accordingly, a HbA1c level ≥ 6.5% (≥48 mmol/mol) will lead to a DM diagnosis; a level situated between 5.7–6.4% (39–46 mmol/mol) will place patients in the prediabetic category [1,2,4].

In the clinical field, HbA1c is the most studied and used type of glycated hemoglobin; it is identified D-glucose attached to one or both N-terminal valines belonging to the β-chain of hemoglobin. This attachment is achieved through a glycation process dependent on the concentration of glucose to which the erythrocytes are exposed [5,6]. The protein level reflects the average blood glucose concentration over a period of 8–12 weeks, a concentration directly proportional to the average lifetime of erythrocytes [7]; this statement is valid only if the blood glucose concentration as well as the life span of erythrocytes have been stable during 3–4 months preceding the time when HbA1c was tested [8]. Conditions associated with erythrocyte instability, such as in hemolytic anemia, splenectomy, liver diseases, chronic kidney and chronic opioid use should be identified and taken into consideration as influencing factors of the true positive rates and should be considered as exclusion criteria when measuring postmortem HbA1c levels with the purpose of identifying DM [8,9,10].

In forensic medicine, the postmortem determination of HbA1c represents a helpful tool in identifying undiagnosed or poorly glycemic controlled diabetes, as well in differentiating diabetic ketoacidosis from alcoholic ketoacidosis [11,12]. It is stated that HbA1c corresponds well to antemortem values, is reasonably stable after death and it shows the smallest deviation from healthy subjects [6,13,14]. Moreover, different authors claim that HbA1c is stable after death for at least 36 h and it remains unaffected by hemolysis [13,15,16,17]. Although the importance of determining this biomarker is acknowledged, there is still no uniformity in the usage of thanatochemistry across forensic services. HbA1c is determined only in a number of centers, without this determination constituting a practice in all the forensic services of Romania.

The purpose of this study is to determine postmortem the glycated hemoglobin (HbA1c) biomarker using a point-of-care analyzer, in order to confirm the antemortem diabetes status in patients with a documented history of DM, and to evaluate the potential use of this method in identifying undiagnosed pre-mortem people with diabetes as well as those with inadequate glycemic control. Through this research, we want to emphasize the value of thanatochemistry and to contribute to the solidification and application of this domain in the field of forensic medicine, worldwide but especially nationally.

Also, our study focuses on comparing the measured values of HbA1c obtained postmortem in two different sampling sites (central versus peripheral blood), for assessing which one is more reliable in postmortem determination of a potential diabetes status.

## 2. Materials and Methods

This study was conducted in accordance with the standards developed by the Declaration of Helsinki, the approval of the management of the Institute of Forensic Medicine Iași and also in accordance with the standards developed by the Research Ethics Commission of the University of Medicine and Pharmacy “Grigore T. Popa”, Iași, Romania (no. 7244/12 April 2018).

### 2.1. Autopsy Data

Within this study a total number of 90 forensic cases were analyzed, with autopsies being performed at the Institute of Forensic Medicine Iași, Romania. The cases included mainly sudden deaths at home, as well as in-hospital deaths that were considered forensic cases. According to the Romanian legislation, a forensic autopsy is ordered in case of violent death, suspicious death, when the cause of death is not known or when there is a reasonable suspicion that a crime might have been committed [18]. All cases of violent death were excluded, such as the cases where there was a suspicion of intoxication. The cases presenting iron deficiency anemia, splenectomy, pregnancy or polycythemia were also excluded; this was done given that such elements could have interfered with HbA1c levels. The autopsies were performed at a postmortem interval of 24–72 h. The antemortem diagnosis of diabetes was established, in 13 out of the 90 cases, based on investigation data, history or medical documents. Out of these 13 cases, only one case was classified as type I DM, the rest being type II. The age of the subjects varied from 30 to 99 years (M = 61.53, SD = 15.05); 29 of them were females (32.22%) and 61 males (67.78%).

### 2.2. Blood Samples

Two blood samples were collected for each case—peripheral and central. The peripheral blood was collected from the femoral vein, identified in the Scarpa’s triangle. The central blood was obtained from the right and left heart cavities, immediately after sectioning the walls of the heart parallel to the interatrial and interventricular septum. The blood was collected in 6 mL vacuum flasks with sodium fluoride (Na Fl) and stored immediately after collection, in the refrigerator, at a temperature of 4 °C, until the end of the autopsy; immediately after its end the blood was processed. Hence, our study comprises a total number of 180 HbA1c values, 90 determinations from the peripheral blood and 90 from the central blood.

### 2.3. The HbA1c Method

The determination of HbA1c was performed by means of a fully automatic analyzer (HemoCue HbA1c 501), acquired for the doctoral research programs. The analyzer was certified by the National Glycohemoglobin Standardization Program (NGSP)/Diabetes Control and Complications Trial (DCCT) and calibrated according to the standards developed by the International Federation of Clinical Chemistry (IFCC). The method principle was based on the fully automated boronate affinity assay, with the percentage determination of HbA1c ranging between 4.0–14.0% (NGSP), the result being obtained in 5 min. The amount of blood sample required is of only 4 μL and may be of capillary or venous origin. The calibration of the device was performed daily and monthly, using two types of cartridges, provided by the manufacturer and, of course, before the expiration date.

It is worth mentioning that, for reinsurance purposes regarding the quality of the results, in the first 10 cases, HbA1c was also determined using the HPLC (High-Performance Liquid Chromatography assay) method, by transporting and processing the samples, for a fee, to a local medical analysis laboratory.

Also, to evaluate the reliability and repeatability of the HbA1c results obtained using the automatic point-of-care system mentioned above, for the first ten autopsy cases, HbA1c values were determined twice in both peripheral and central blood. The second determination of HbA1c from the same blood sample (peripheral and central, respectively) was identical each time. Thus, we concluded that it was no longer necessary to continue the verification for the other 80 cases, taking into account the same inclusion criteria and the same sampling conditions.

### 2.4. Reference Values and Classification of HbA1c Values in the Diagnosis of DM

Currently, according to the ADA and the WHO criteria, specific HbA1c values can determine the following different glycemic conditions: normal 4.8–5.6%; prediabetes 5.7–6.4%; diabetes ≥ 6.5%. The therapeutic target for diabetics is of 7% [1,2]. We applied the ADA/WHO criteria on our postmortem HbA1c measured values, in order to stratify the cases accordingly.

### 2.5. Statistical Analysis

The data was analyzed using SPSS version 26 software (IBM, Armonk, NY, USA)—and Excel 2016 version 16.0 software (Microsoft, Redmond, WA, USA) functions (mean, median function, Goodman and Kruskal’s Gamma Coefficient). The normal distribution of HbA1c values was assessed using the Shapiro-Wilk W Test. Results were shown as percentages, as mean ± standard deviation, and were applicable as median, maximum/minimum values and inter-quartile range.

## 3. Results

From the 90 autopsy cases, a total number of 180 HbA1c values were assessed, 90 determinations being from the peripheral blood and 90 from the central blood.

The interpretation of the 90 determinations of HbA1c from the peripheral blood, based on the reference values, led to the following division of the study lot: 49% (44 cases) had values within the normal range, 26% (23 cases) were classified as prediabetic condition and 25% (23 cases) were classified as diabetes.

Also, applying the reference values to all of the 90 determinations of HbA1c sampled from the central blood, the following classification was obtained: 59% (53 cases) had values within the normal range, 21% (19 cases) were classified as prediabetic status and 20% (18 cases) were classified as diabetes.

The percentage of patients identified postmortem with altered glycemic status (cases that had HbA1c values equal to or greater than 5.7%) was as follows: 51% demonstrable by peripheral blood determinations and 41% demonstrable by central blood determinations.

All 13 cases antemortem diagnosed with DM were confirmed postmortem by HbA1c values obtained from both peripheral and central blood samples (as shown in Table 1). By applying the same ADA/WHO criteria, the postmortem HbA1c values blood samples, revealed new cases of DM that weren’t antemortem diagnosed: 10 cases from the peripheral blood and 5 cases from central blood.

The levels of HbA1c from peripheral blood samples were correlated with the HbA1c values obtained from central blood.

To this effect, HbA1c values ranging in normal limits (up to 5.6%), were coded with code 1, code 2 indicating prediabetes (between 5.7–6.4%) and values coded as 3 indicating diabetes (with values of 6.5% and above). The Gamma correlation coefficient was calculated as G = 0.877 and indicated a high correlation between the evaluation of HbA1c in peripheral blood and that in central blood.

Given that in a normal distribution the values of Skewness and Kurtosis are equal to zero, the obvious deviations of these parameters as indicated in Table 2, suggest that both HbA1c values, peripheral and central, are not distributed along a normal curve. As such, the distribution of HbA1c values in the blood (peripheral and central) is characterized by the presentation of median value, minimum and maximum values and the interval between quartiles as represented in Table 3.

**Figure 1:**
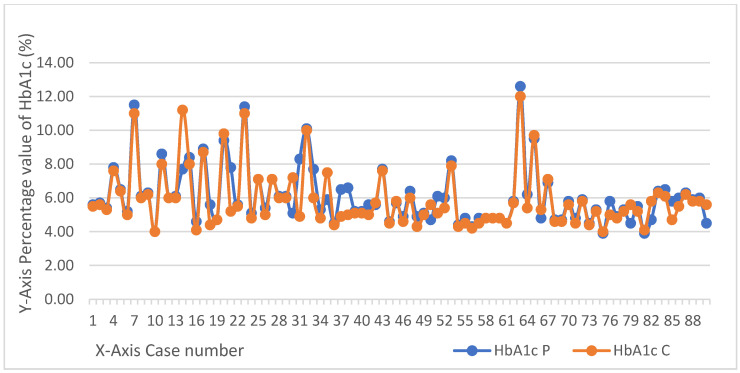
Curves HbA1c values in peripheral (HbA1c P) and central (HbA1c C) blood.

The measured values of HbA1c in postmortem blood samples, peripheral and central, are depicted in Figure 1.

In terms of age, most cases with a postmortem DM diagnosis, determined using HbA1c, from both peripheral and central blood, were framed in the sixth and seventh decades of age. The predominant gender was male (67.78%).

## 4. Discussion

DM represents a major health problem, with a negative impact on the quality of life, thus the necessity of studying it at all possible levels. Although carbohydrate metabolism disorders are intensively studied during the life-time of a patient, with biochemical parameters being easily determined, with clear reference intervals, validated and respected worldwide, their postmortem analysis seems to be located at the exact opposite pole.

HbA1c is an essential parameter in diabetic pathology, which, unlike glucose, remains stable postmortem [19,20,21]. Thus, our study, centered on determining the levels of HbA1c in necropsy blood samples taken from different sampling sites (peripheral and central), underlines the importance and the value of this biomarker in forensic practice and the acute need of integrating this parameter in routine thanatochemistry protocols.

By applying the ADA (American Diabetes Association) and the WHO (World Health Organization) criteria on our postmortem HbA1c levels, our study revealed a significant number of patients with altered glycemic statuses which had remained undiagnosed during their lifetime: 51% demonstrable by peripheral blood determinations and 41% demonstrable by central blood determinations. 23 cases with DM (25%) were identified by analyzing the peripheral blood, and 18 cases with DM (20%) were identified by analyzing the central blood. However, only 13 cases out of the total number of examined 90 cases presented an antemortem diagnosis of DM. The fact that all 13 cases diagnosed antemortem with DM, were confirmed postmortem in both peripheral and central blood samples, suggests validation of the method applied in our study. Moreover, our additional findings (10 more diagnosed postmortem DM cases using peripheral blood levels of HbA1c and 5 more using central blood levels of HbA1c) emphasize the importance of determining and interpreting postmortem HbA1c values, especially in deceased patients with no data or information leading to a possible imbalance of carbohydrate metabolism during lifetime.

DM is underdiagnosed worldwide and therefore the application of thanatochemistry, through systematic determinations in forensic services, at least in cases of sudden death, can bring real benefits in this regard.

As recommended by the ADA [4], it is necessary to verify the diagnosis of DM, by performing two determinations for one or two of the specific biochemical parameters (HbA1c, fasting blood glucose, glucose tolerance test), which exceed the diagnostic threshold values. This is why, in our study, we aimed to obtain two different determinations of the most stable postmortem biomarker (HbA1c), harvested from two different sites—the peripheral part and the central part.

Analyzing the HbA1c for the same case, we observed a slight difference between the values obtained from the two different sites. These differences, that depend on the place of harvest, must be known and identified, especially when only a certain type of harvest is possible, either central or peripheral. It goes without saying that this kind of examination can only be performed postmortem and never while the patient is still alive. There are researchers who claim that any of the above sites can be used because there are no significant differences among them [19,21,22]; they do however claim that the value in peripheral blood has a statistically lower value [17]. In our research we also found that there is a very strong correlation between the two sampling sites. However, by comparing the median value for the two sites, we found that the value of HbA1c from the peripheral blood has a statistically higher value, being therefore in discordance with the results of the published studies mentioned above [22]. A possible explanation of this finding could be the use of a certain type of analyzer; but even so, the differences are often insignificant, an aspect also stated by other researchers [19,21,22].

We’ve analyzed the differences between the HbA1c levels of the same case measured both in peripheral and central blood and noticed a higher difference (up to 3.5%) in the cases autopsied after 72 h postmortem. Smaller differences were found in cases where the autopsy was performed closer to the time of death (postmortem interval between 24–36 h). The main explanation for these differences can be attributed most likely to postmortem changes, as stipulated in others studies [13,15,16,17].

Subsequently, another question that could be answered analyzing the collected data, is which of the two sampling sites has a higher chance in confirming the antemortem diabetic status. Overall, the data indicated in Table 1 and Figure 1, regarding the subjects with antemortem diagnosis of diabetes (13 in total), showed that both measurements from the selected sampling sites were in agreement with the antemortem diabetic status. Moreover, higher concentrations of HbA1c were detected in peripheral blood samples compared to central blood samples (10 cases out of 13 showed higher values). These results suggests that this site could be more reliable for sampling when dealing with cases with antemortem DM confirmed status.

Our results showed that the HbA1c levels from all the peripheral blood were able to detect five more cases of DM than the ones sampled from the central blood. Even though our research excluded from the study lot cases presenting possible interferences with postmortem HbA1c levels (e.g., iron deficiency anemia, splenectomy, pregnancy or polycythemia) and also identified in peripheral blood samples a larger number of new cases (compared with central blood samples) with high HbA1c levels that could be classified as ”postmortem” DM, still we cannot state for sure that the peripheral site can be considered the optimal site for sampling. Due to the small size of the study lot, and the fact that not all of our samples were processed twice or double checked with a second automated method (e.g., HPLC technique, turbidimetry) [21], thus eliminating potential false positive results, future investigation is necessary for confirming this supposition.

Another aspect worth mentioning is the high levels of HbA1c in people with diabetes identified postmortem. There are studies that show the close association of high HbA1c levels in diabetics with the occurrence of acute cardiovascular events [23,24]. We believe that this postmortem-determined biochemical marker could fall within the parameters that are to be identified when a cardiovascular thanatogenic mechanism is suspected, especially among people with diabetes.

The advantages of using this analyzer are the easy and clean technique which can process a small amount of samples and the rapid determination of the value of HbA1c. This is the first thanatochemical study that was carried out with this type of analyzer and it appears to have provided feasible postmortem results. Although it has an international approval and certification, complementary studies in the field of forensic medicine are needed, implying the determination of HbA1c on a larger number of cases, over longer periods of time and applied in multiple forensic centers.

## 5. Conclusions

It is known that many people with diabetes remain undiagnosed. That is why it is necessary to continue postmortem investigations, with the identification and diagnosis of diabetes even after death. HbA1c remains the most stable biomarker analyzed postmortem on which a coroner can rely in order to identify a possible imbalance in glucose metabolism, or even to diagnose diabetes. Our study managed to confirm the antemortem diagnosis of DM using a simple point-of-care analyzer and applying standardized and certified criteria on HbA1c levels measured postmortem. We also identified a considerable number of cases which could be classified as DM applying ADA/WHO criteria, in patients with no antemortem history of glucose imbalance—at least 20% more cases. Although the two different sites used for blood collection showed a strong statistical correlation, it seems that the peripheral site could have a higher reliability in postmortem confirmation of a diabetic status evaluated during lifetime.

We believe that our research reveals the importance of knowing the postmortem applicability of HbA1c.It also provides new information and suggests new paths for future research in the field of thanatochemistry.

## Figures and Tables

**Table 1:** Antemortem diagnosed DM cases with corresponding postmortem measured glycated hemoglobin (HbA1c).

Cases with Antemortem Diagnosis of DM	Case 1	Case 2	Case 3	Case 4	Case 5	Case 6	Case 7	Case 8	Case 9	Case 10	Case 11	Case 12	Case 13
Postmortem interval of sampling	36 h	72 h	24 h	24 h	24 h	24 h	24 h	24 h	24 h	24 h	36 h	24 h	24 h
HbA1c concentration in peripheral blood	8.6%	7.7%	8.4%	8.9%	9.4%	11.4%	7.1%	7.1%	10.1%	7.7%	12.6%	9.5%	6.9%
HbA1c concentration in central blood	8%	11.2%	8%	8.7%	9.8%	11%	7.1%	7.1%	10%	7.6%	12%	9.7%	7.1%

**Table 2:** Results of testing the normal distribution of HbA1C values in peripheral and central blood.

HbA1c	*N*	Skewness	Std. Err. Skewness	Kurtosis	Std. Err. Kurtosis
Peripheral blood	90	1.69	0.25	3.21	0.50
Central blood	90	1.80	0.25	3.01	0.50

**Table 3:** Statistical distribution of measured HbA1c values in both peripheral and central blood samples.

HbA1c	*N*	Median	Minimum	Maximum	Lower Quartile	Upper Quartile
Peripheral blood	90	5.70	3.9	12.6	4.8	6.5
Central blood	90	5.45	4.0	12.0	4.8	6.1

## Data Availability

The study did not report any data, apart from those reported already in the article.
